# Exercise-Induced Alterations in Skeletal Muscle, Heart, Liver, and Serum Metabolome Identified by Non-Targeted Metabolomics Analysis

**DOI:** 10.3390/metabo7030040

**Published:** 2017-08-08

**Authors:** Joseph W. Starnes, Traci L. Parry, Sara K. O’Neal, James R. Bain, Michael J. Muehlbauer, Aubree Honcoop, Amro Ilaiwy, Peter M. Christopher, Cam Patterson, Monte S. Willis

**Affiliations:** 1 Department of Kinesiology, University of North Carolina at Greensboro, Greensboro, NC 27412, USA; jwstarne@uncg.edu (J.W.S.); petechristopher86@gmail.com (P.M.C.); 2 McAllister Heart Institute, University of North Carolina, Chapel Hill, NC 27599-7525, USA; Traci_parry@med.unc.edu (T.L.P.); 3 Department of Pathology & Laboratory Medicine, University of North Carolina, Chapel Hill, NC 27599-7525, USA; 4 Sarah W. Stedman Nutrition and Metabolism Center, Duke Molecular Physiology Institute, Duke University Medical Center, Durham, NC 27708, USA; sara.oneal@duke.edu (S.K.O’N.); james.bain@duke.edu (J.R.B.); amroilaiwy@gmail.com (A.I.); 5 Division of Endocrinology, Metabolism, and Nutrition, Department of Medicine, Duke University Medical Center, Durham, NC 27703, USA; michael.muehlbauer@duke.edu; 6 Toxicology Curriculum, University of North Carolina, Chapel Hill, NC 27599, USA; n18d478@email.unc.edu (A.H.); 7 Presbyterian Hospital/Weill-Cornell Medical Center, New York, NY 10065, USA; cpatters@nyp.org; 8 Department of Pharmacology, University of North Carolina, Chapel Hill, NC 27599, USA

**Keywords:** exercise, metabolism, skeletal muscle, heart, liver, serum, non-targeted metabolomics

## Abstract

Background: The metabolic and physiologic responses to exercise are increasingly interesting, given that regular physical activity enhances antioxidant capacity, improves cardiac function, and protects against type 2 diabetes. The metabolic interactions between tissues and the heart illustrate a critical cross-talk we know little about. Methods: To better understand the metabolic changes induced by exercise, we investigated skeletal muscle (plantaris, soleus), liver, serum, and heart from exercise trained (or sedentary control) animals in an established rat model of exercise-induced aerobic training via non-targeted GC-MS metabolomics. Results: Exercise-induced alterations in metabolites varied across tissues, with the soleus and serum affected the least. The alterations in the plantaris muscle and liver were most alike, with two metabolites increased in each (citric acid/isocitric acid and linoleic acid). Exercise training additionally altered nine other metabolites in the plantaris (C13 hydrocarbon, inosine/adenosine, fructose-6-phosphate, glucose-6-phosphate, 2-aminoadipic acid, heptadecanoic acid, stearic acid, alpha-tocopherol, and oleic acid). In the serum, we identified significantly decreased alpha-tocopherol levels, paralleling the increases identified in plantaris muscle. Eleven unique metabolites were increased in the heart, which were not affected in the other compartments (malic acid, serine, aspartic acid, myoinositol, glutamine, gluconic acid-6-phosphate, glutamic acid, pyrophosphate, campesterol, phosphoric acid, creatinine). These findings complement prior studies using targeted metabolomics approaches to determine the metabolic changes in exercise-trained human skeletal muscle. Specifically, exercise trained vastus lateralus biopsies had significantly increased linoleic acid, oleic acid, and stearic acid compared to the inactive groups, which were significantly increased in plantaris muscle in the present study. Conclusions: While increases in alpha-tocopherol have not been identified in muscle after exercise to our knowledge, the benefits of vitamin E (alpha-tocopherol) supplementation in attenuating exercise-induced muscle damage has been studied extensively. Skeletal muscle, liver, and the heart have primarily different metabolic changes, with few similar alterations and rare complementary alterations (alpha-tocopherol), which may illustrate the complexity of understanding exercise at the organismal level.

## 1. Introduction

Exercise therapies for both the prevention and treatment of cardiovascular disease have proven beneficial over decades of research [1,2]. The metabolic and physiologic responses to exercise have been increasingly of interest [3,4], with regular physical activity enhancing antioxidant capacity, exhibiting improvement in cardiac function, the offering protection against the development of chronic diseases such as type 2 diabetes, cardiovascular disease, cancer, hypertension, neurodegeneration, and aging [5,6,7,8,9,10,11,12,13,14].

Multiple studies have investigated the metabolic adaptations during exercise training [15,16,17]. Recent studies have explored the skeletal muscle metabolome to identify metabolic signatures in biopsies from exercise trained humans [18]. Alterations in branched chain amino-acids and genes related to tissue remodeling were identified, with modest changes in plasma metabolite levels [18]. Other studies have identified metabolic alterations in the liver, a critical component of energy metabolism during exercise [19]. No studies to date have investigated the overall system in which these tissues likely to “talk” to each to carry out the intricate and far reaching exercise-induced adaptations.

In the present study, we sought to identify the exercise-induced alterations in multiple muscle types (plantaris, soleus), liver, serum, and heart from the same animals using non-targeted GC/MS methodologies in an established rat model of moderate-intensity aerobic exercise training [20,21]. A primary reason for this is to better understand the cross-talk between tissues, which is critical in making systematic adaptations to exercise. Because of the critical cross-talk between tissues, predicting system-wide effects on a particular system (e.g., the cardiovascular components) is challenging and requires a more holistic approach to identifying how the metabolic components of different tissues are affected by exercise in vivo. In the present study, we identify specific metabolic alterations that are common between compartments, yet distinctly different between tissues, illustrating the minimal alterations seen circulating in serum at the end of the training period.

## 2. Results

### 2.1. Body and Muscle Weights

Body and muscle weights of sedentary and exercise-trained animals are displayed in Table 1. The exercise training protocol did not affect absolute heart weight, but it resulted in a modest increase (7.7%) in heart weight-to-body weight ratio (*P* < 0.05). No exercise-induced hypertrophy was observed for soleus or plantaris muscles, which is consistent with our earlier endurance exercise studies [22].

### 2.2. Plantaris Muscle

**Figure 1:**
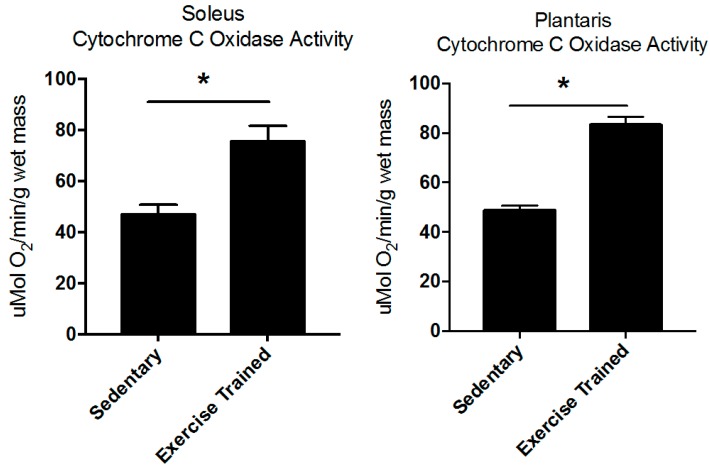
Exercise-induced increases in soleus and plantaris cytochrome c oxidase activity. Total Cytochrome *c* oxidase activity in sedentary and exercise trained rats (*N* = 9–10/group). * *P* < 0.05 vs. sedentary. Values are expressed as mean values ± SE (*N* = 9–10 muscles/group).

**Figure 2:**
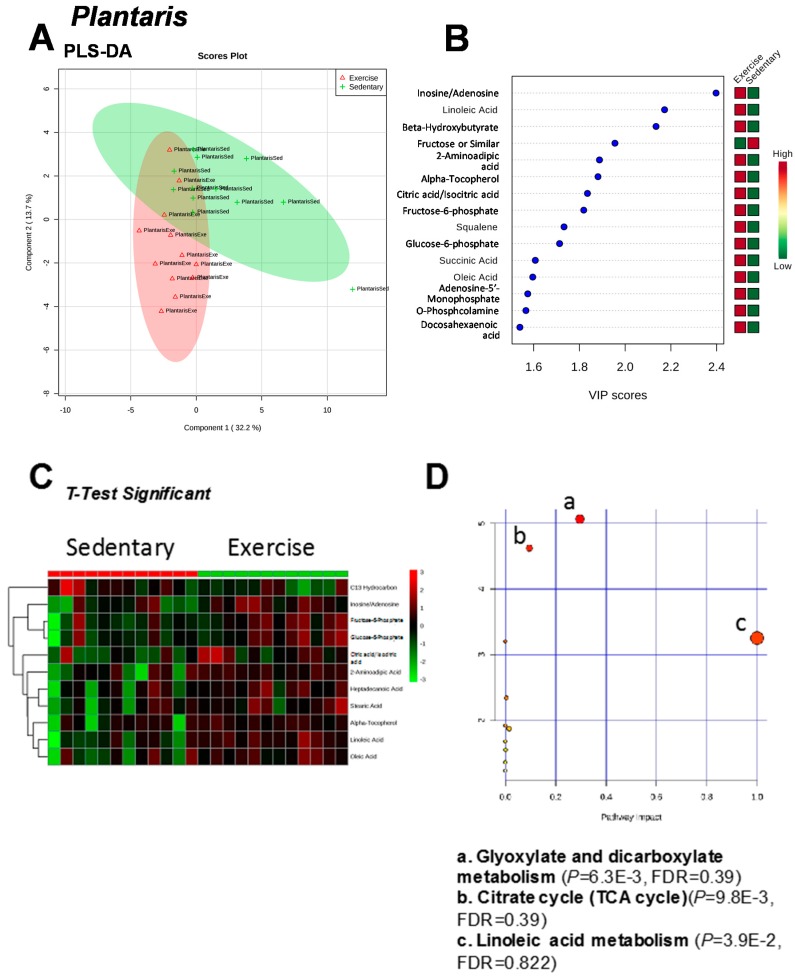
Analysis of non-targeted metabolomics of plantaris muscle from exercise-trained and sedentary control rats. (**A**) Partial Least Squares Discriminant Analysis (PLS-DA). (**B**) PLS-DA Variable Importance in the Projection (VIP) significant metabolites. (**C**) *t*-Test significant metabolites (*P* < 0.05). (**D**) Pathway analysis based on t-test significant metabolites. *N* = 12/group.

**Figure 3:**
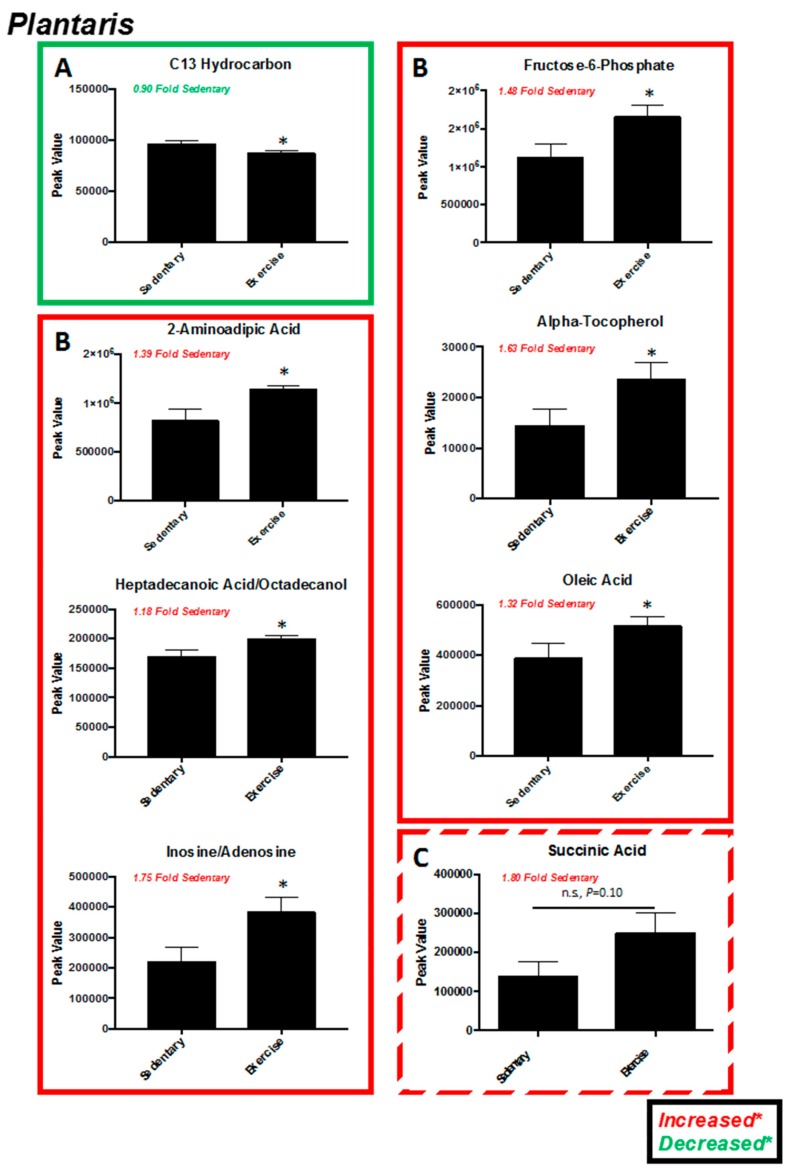
*t*-Test significant metabolites and succinic acid from plantaris muscle. (**A**) Metabolite decreased in exercise-trained muscle. (**B**) Metabolites increased in exercise-trained muscle. (**C**) Metabolite increased in exercise-trained muscle (not significant). Data represent mean ± SEM. * *P* < 0.05. *N* = 12/group.

**Figure 4:**
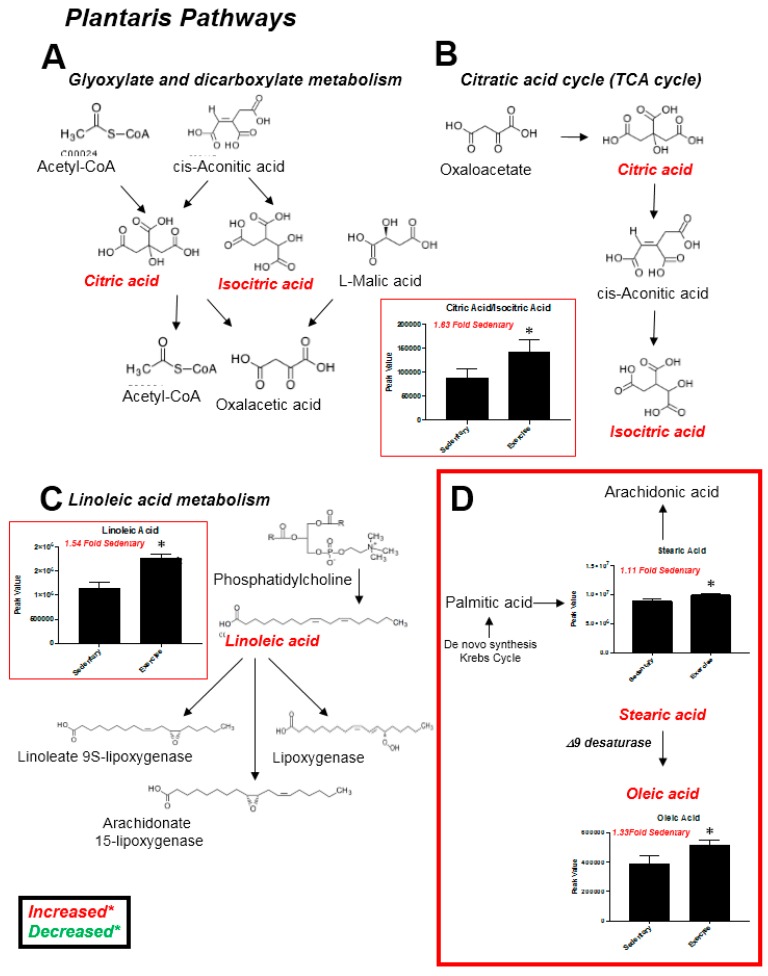
Pathway analysis of *t*-test significant metabolites. (**A**) Glyoxylate and dicarboxylate metabolism. (**B**) Citric acid (TCA) cycle. (**C**) Linoleic acid metabolism. (**D**) Long-chain fatty acid synthesis. Data represent mean ± SEM. * *P* < 0.05. *N* = 12/group.

Exercise training five days a week on a motor-driven treadmill resulted in significant increases in soleus and plantaris muscle cytochrome *c* oxidase activity, evidence of exercise-induced mitochondrial biogenesis (Figure 1). Eighty-five metabolites were identified in plantaris muscle, which overlapped on principal component (PC) 1, but separated to some degree along PC 2 (Figure 2A, Figure S1). Using the variable interdependent parameters (VIP) analysis, three metabolites were >2 fold (inosine/adenosine, linoleic acid, and beta-hydroxybutyrate) (Figure 2B). Eleven metabolites were significant by *t*-test (C13 hydrocarbon, inosine/adenosine, fructose-6-phosphate, glucose-6-phosphate, citric acid/isocitric acid, 2-aminoadipic acid, heptadecanoic acid, stearic acid, alpha-tocopherol, linoleic acid, and oleic acid (Figure 3C). Pathway analysis of these eleven metabolites identified enrichment for the glyoxylate and dicarboxylate metabolism (*P* = 6.3 × 10^−3^, FDR = 0.39, Figure 2D). Of these eleven metabolites, 10 were increased, with the exception of the C13 hydrocarbon (Figure 3). In the glycoxylate and dicarboxylate metabolic pathway (Figure 4A) and the citric acid cycle (Figure 4B), citric acid/isocitric acid was significantly increased. Additionally, increases in linoleic acid (Figure 4C) were identified, along with stearic acid and oleic acid (Figure 4D).

### 2.3. Liver

**Figure 5:**
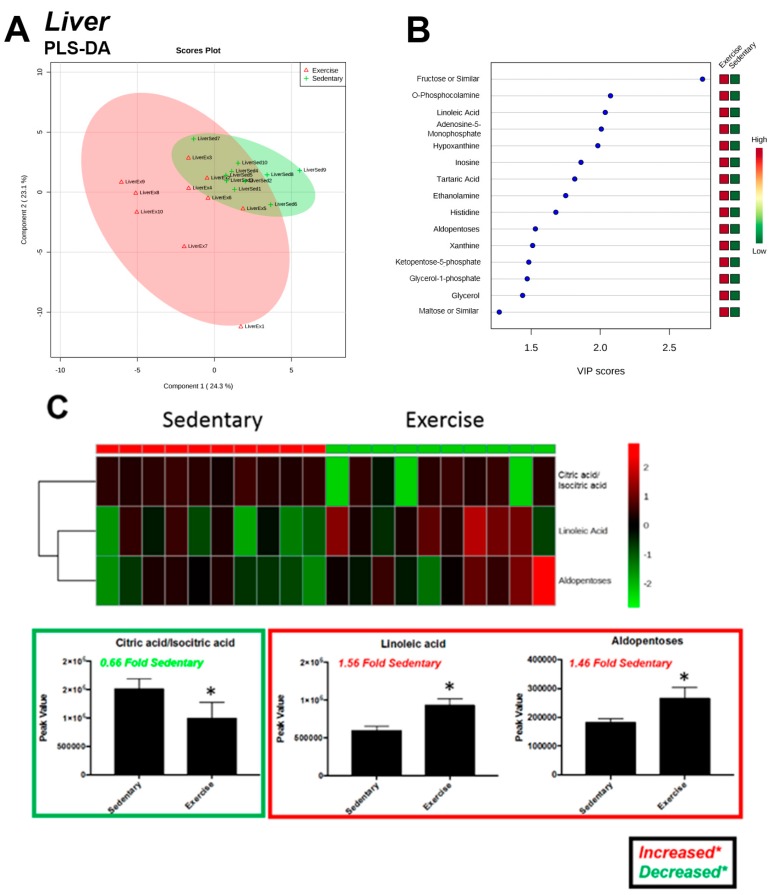
Analysis of non-targeted metabolomics of liver from exercise-trained or sedentary control rats. (**A**) Principal components analysis using PLS-DA. (**B**) Variable importance in projection (VIP) scores. (**C**) Heatmap of *t*-test significant liver metabolites in exercise-trained rats vs. sedentary. Data represent mean ± SEM. * *P* < 0.05. *N* = 12/group.

**Figure 6:**
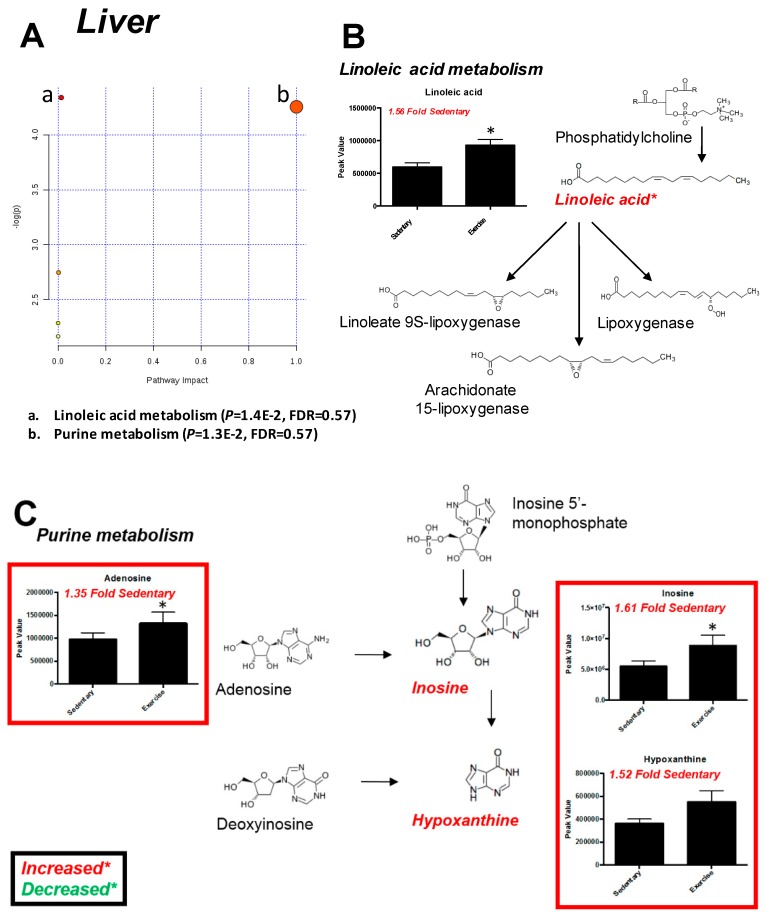
Pathway analysis of *t*-test significant liver metabolites from exercise-trained vs. sedentary control rat livers. (**A**) Pathway analysis based on *t*-test significant metabolites. (**B**) *t*-Test significant metabolite related to linoleic metabolism. (**C**) *t*-Test significant metabolite related to purine metabolism. Data represent mean ± SEM. *P < 0.05. *N* = 12/group.

Eighty-two metabolites were identified in the liver, with considerable overlap in both PC1 and PC2, illustrating considerable commonality on the whole in the liver metabolites identified in exercise trained rats vs. sedentary controls (Figure 5A, Figure S2). By VIP analysis, only one metabolite was >2.0 (Fructose or Similar) (Figure 5B), with three metabolites significant by *t*-test (Figure 5C). Citric acid/isocitric acid was decreased to 0.66 fold sedentary levels, while linoleic acid and aldopentoses were increased ~1.5 fold sedentary liver levels (Figure 5C). Pathway analysis of *t*-test and VIP significant metabolites (fructose, linoleic acid, hypoxanthine, inosine, ketopentose-5-phosphate, aldopentoses) identified linoleic and purine metabolism involvement (Figure 6A). Liver linoleic acid was 1.56 fold sedentary levels after exercise training (Figure 6B). Purine metabolism involvement was suggested by VIP analysis, including increased adenosine (1.35 fold sedentary), inosine (1.61 fold sedentary), and hypoxanthine (1.52 fold sedentary) (Figure 6C).

### 2.4. Soleus and Serum

Analysis of the soleus muscle after exercise training identified 72 metabolites, with considerable overlap in metabolic profiles (Figure S3A). VIP analysis identified four metabolites with scores >2.0 (squalene, *O*-Methylphosphate, Glycine, and Glucose-6-phospate (Figure S3B). Only one *t*-test significant metabolite was identified in soleus (glycine, *P* = 3.9 × 10^−2^, FDR = 9.8 × 10^−1^) (Figure S3C). Like the soleus muscle, analysis of serum found that the metabolic profiles in exercise trained and sedentary were largely overlapping (Figure S4A). Of the 48 metabolites identified in serum, none were found to have VIP scores >2 (Figure S4B) and only one *t*-test significant metabolite was found in the serum (alpha-tocopherol, *P* = 4.0 × 10^−2^, FDR = 6.8 × 10^−1^) (Figure S4C). With only one metabolites significant in the soleus and serum, little insight into the affected pathways are known (Figure S5).

### 2.5. Heart

Seventy-seven metabolites were identified in the heart (Figure S6), with some overlap by PLS-DA principal components (Figure S7A). Two metabolites had VIP scores >2.0 (serine and glutamine, Figure S7B), with eleven *t*-test significant (Figure S7C). Pathway analysis identified alanine, aspartate, and glutamate metabolism, methane metabolism, and aminoacyl-tRNA biosynthesis as involved (Figure S7D), with significant increases in aspartic acid and glutamic acid, with significantly decreased glutamine in the heart with exercise (Figure S8). All other *t*-test significant metabolites were increased (pyrophosphate, myoinositol, malic acid, creatinine, phosphoric acid, and campesterol, Figure S9).

### 2.6. Exercise-Induced Changes across Plantaris, Liver, Soleus, Serum, and Heart

Exercise-induced alterations in metabolites varied across tissues, with the soleus and serum affected the least (Table 2). The alterations in the plantaris muscle and liver were most alike, with four (4) metabolites increased in each (citric acid/isocitric acid, inosine/adenosine, fructose/fructose-6-phosphate, and linoleic acid) (Table 2). Similarly, fructose-6-phosphate was increased in the heart (Table 2). Significant increases in alpha-tocopherol in the plantaris muscle paralleled decreased serum alpha-tocopherol (Table 2). The heart had increases in twelve metabolites, eleven (11) which were not affected in the other compartments (Table 2).

## 3. Discussion

In the present study, we investigated the plantaris and soleus muscles in rats using a non-targeted metabolomics analysis after a progressive moderate-intensity training regimen corresponding to 75–80% VO_2max_ (maximum volume of oxygen consumed, mL/kg/min) for 6–7 weeks. We identified significant alterations in exercise trained muscle compared to sedentary controls related to glyoxylate metabolism, citric acid metabolism, and linoleic metabolism (Figure 4). These findings complement prior studies using targeted metabolomics approaches to determine the metabolic changes in exercise-trained human skeletal muscle [23]. In response to training, vastas lateralis muscle was found to have increases in acyl-carnitines (primarily medium- and long-chain species generated as beta-oxidation byproducts) [23]. Interestingly, the C18:2, C18:1, and C18:0 (corresponding to linoleic acid, oleic acid, and stearic acid, respectively) were increased with all the exercise training regimens except the lowest intensity of training (low aerobic/resistance training) compared to the inactive groups [23]. In addition, exercise-induced increases in succinate were identified in all of the exercise training regimens except the lowest training intensity (low aerobic/resistance training), along with increases in citric acid at the highest intensity training regimen [23]. These parallel the findings in the present study, where we identified significant increases in linoleic, oleic, and stearic acid (summarized in Table 2). Significant increases in plantaris citric acid were identified (Figure 4A,B), while increases in succinic acid were identified, but did not reach significance (*P* = 0.10) (Figure 3B, lower right corner). The present study additionally identified increases in plantaris alpha-tocopherol (also known as vitamin E) after exercise training, along with inosine/adenosine, glucose-6-phsophate, 2-aminoadipic acid, and heptadecanoic acid (Table 2). The findings from these two studies demonstrate the utility of both the targeted metabolomics approach, allowing a more detailed description of metabolic pathways (acyl-carnitines and TCA cycle in the prior human study of vastus lateralis [23]), and the broader novel findings outside of the acyl-carnitines/TCA cycle (e.g., significant increases in muscle inosine/adenosine, glucose-6-phsophate, 2-aminoadipic acid, and heptadecanoic acid) in the present study using a non-targeted metabolomics approach.

While increases in alpha-tocopherol have not been identified in muscle after exercise to our knowledge, the benefits of vitamin E (alpha-tocopherol) supplementation in attenuating exercise-induced muscle damage has been studied extensively [24]. While anti-oxidants such as vitamin E have been hypothesized to improve skeletal muscle contractile performance, this is based on the rapid elevation of oxidant concentration during exercise as a contributory factor [6,25]. Others have found that anti-oxidant supplementation can blunt biochemical adaptations to exercise [26,27]. In the present study, we also identified that the only significantly altered serum metabolite was also alpha-tocopherol (Table 2). Interestingly, the increases in plantaris alpha-tocopherol parallel significant DECREASES in serum alpha-tocopherol, suggesting a dynamic whereby alpha-tocopherol moves to the muscle in order to assist in the adaptation to exercise-induced stress. These adaptations include the repair of the plasma membrane in skeletal muscle [28,29].

Exhaustive exercise and endurance training also produces changes in liver metabolism. In a recent study of Sprague-Dawley rats randomized to sedentary, exhaustive (final speed of 30 m/min to exhaustion), or endurance training (3% gradient, 5 days/week, 12 weeks), the liver was analyzed using a targeted metabolomics approach to identify the changes that occur with exercise [19]. These studies identified that endurance training significantly increased liver alanine, glycine, threonine, glutamine, lactate, succinate, fumarate, malate cysteine, ornithine, β-aminoisobutyric acid, aminomalonic acid, ascorbic acid, and gluconic acid, while reducing the concentration of liver arachidonic acid, reflecting increases in liver TCA- and Urea-cycle activities [19]. In the present study, all of these metabolites were identified except cysteine, β-aminoisobutyric acid, and aminomalonic acid (not reaching statistical significance). Most were clearly not increased (liver alanine 1.08 fold sedentary, glycine 1.07 fold sedentary, glutamic acid 0.91 fold sedentary, malic acid 0.99 fold sedentary, ornithine 0.79 fold sedentary), while others were increased without significance (lactic acid 1.16 fold sedentary, succinic acid 1.37 fold sedentary, gluconic acid 1.23 fold sedentary). We uniquely identified significant increases in linoleic acid, and metabolites involved in purine metabolism (adenosine, 1.35 fold sedentary, inosine, 1.61 fold sedentary, and hypoxanthine, 1.52 fold sedentary, Figure 6C).

## 4. Materials and Methods

### 4.1. Animals and Exercise Treatment

Male Sprague-Dawley rats were purchased from Charles River and housed in the UNC-Greensboro animal facility in an isolated room maintained at 22 °C with a 12:12 h light-dark cycle. They were singly housed to allow for food consumption monitoring. At 8 weeks of age, they were divided into sedentary (S; *N* = 12) and exercise-trained (E; *N* = 12) groups. Exercise was carried out on a motor-driven treadmill, set at a 10.5% incline, 5 days/week for 6–7 weeks in an adjoining room maintained at 20 °C. Running duration and speed were gradually increased over 22 days to 60 min at 30 m/min, corresponding to 75–80% VO_2max_ [30], and then maintained at this level for the remaining 2–3 weeks. We have previously reported that this moderate-intensity aerobic exercise program results in modest cardiac hypertrophy as determined by heart-to-body weight ratio [20,21,31] and a hyperdynamic heart [20] with enhanced protection against ischemia-reperfusion injury [31]. Sedentary rats were placed on a stationary treadmill in the same room during the daily exercise bout. This investigation was approved by the UNC-Greensboro’s Animal Care and Use Committee and conforms to the *Guide for the Care and Use of Laboratory Animals* published by the National Institutes of Health (NIH Publication No. 85–23, Revised 1996).

### 4.2. Tissue and Serum Collection

At least 5 h after the last exercise session, rats were anesthetized with an intraperitoneal injection of pentobarbital sodium (50 mg/kg body wt). Blood was collected from the abdominal aorta under anesthesia. The beating heart was then removed, weighed, and mounted on a Langendorff perfusion apparatus, as previously described [31]. Immediately after heart removal, a second person harvested a small piece of the liver (without perfusion) that was placed directly on dry ice. Within one minute, the soleus and plantaris muscle were removed and also placed immediately on dry ice. The soleus is composed almost entirely of slow twitch oxidative muscle fibers (SO) while the plantaris is almost entirely composed of fast twitch fibers, which are further divided fairly equally into fast glycolytic (FG) fibers and fast oxidative glycolytic (FOG) fibers [32]. The heart was perfused at a perfusion pressure of 60 mm Hg in a non-recirculating mode with oxygenated Krebs-Henseleit buffer (37 °C) containing (in mM) 118 NaCl, 4.7 KCl, 1.25 CaCl_2_, 0.6 KH_2_PO_4_, 1.2 MgSO_4_, 24.7 NaHCO_3_, 10 glucose and 6 IU/L insulin for 10 min to wash out blood then freeze-clamped while beating. Collected blood was incubated 20 min at room temperature to allow clotting then centrifuged at 2000× *g* for 10 min at 4 °C to obtain serum. All tissues and serum were stored at −80 °C until analyzed.

### 4.3. Training Status

Soleus and plantaris muscles from one leg were used for measurement of cytochrome *c* oxidase activity, a marker of mitochondria content. Briefly, muscles were homogenized in 20 volumes of ice-cold 50 mM KH_2_PO_4_, 0.1 mM EDTA, and 0.1% Triton X-100 (pH 7.4), and centrifuged at 10,000× *g* for 5 min at 4 °C. The supernatants were analyzed for cytochrome *c* oxidase activity polarographically at 37 °C using a Clark-type oxygen electrode and done in triplicate as described previously [22].

### 4.4. Non-Targeted Metabolomics Determination by GC–MS Instrumentation

Left ventricular tissue was flash frozen in liquid nitrogen, weighed (25–50 mg wet weight), then placed in buffer (50% acetonitrile, 50% water, 0.3% formic acid) at a standard concentration of 25 mg/475 μL buffer and fully homogenized on ice for 20–25 s. Tissues were then placed on dry ice and stored at −80 °C. Samples were analyzed by GC/MS, as previously described [33]. The raw, transformed, and sorted data used is found in Table S1. Four groups with ten biological replicate samples were analyzed (40 total). If more than 3 individuals did not have a metabolite detected in a group (of 10 total), they were excluded from further analysis for that metabolite. In groups missing values, the lowest value of that group was used to impute those values. The data obtained in this study is freely accessible to the public at the NIH Common Fund’s Data Repository and Coordinating Center (supported by NIH grant, U01-DK097430) website, http://www.metabolomicsworkbench.org, as recently described [34,35,36].

### 4.5. Metabolomic Statistical Analyses

Metaboanalyst (v3.0) run on the statistical package R (v2.14.0) used metabolite peak areas (as representative of concentration) [37,38]. These data were scaled using Pareto scaling feature. For each tissue (and serum), the moderate-intensity aerobic exercise training group was statistically compared to the sedentary group using a *t*-test in Metaboanalyst v3.0. *t*-Test significant metabolites (*P* < 0.05) were used to identify metabolic pathways using the Pathway Analysis and Enrichment Analysis features in Metaboanalyst v3.0. Only metabolites identified and detected in both groups were included in the *t*-test. All data from this study are available in Supplemental Table S1. Data are presented as mean +/− SEM, unless otherwise indicated.

### 4.6. Other Statistical Analyses

Differences between sedentary and exercise-trained groups were compared using an independent *t*-test (2-tailed); and Figure 1 plotted in Prism 7.0 (GraphPad Software, Inc., La Jolla, CA, USA).

## 5. Conclusions

Taken together, our study sheds light on the cross-talk between organs during exercise-induced adaptations. Interestingly, oxidative tissues like the soleus underwent minimal changes (comparing sedentary to endurance exercise trained), while the liver and mixed fast twitch fiber plantaris skeletal muscle exhibited the most extensive exercise-induced adaptations. Both the liver and plantaris showed significant increases in oxidative metabolism. Additionally, increases in metabolites in tissues paralleled decreases of metabolites found in serum. Such data show that exercise mediated adaptations can occur in tissues other than exercising skeletal muscles, indicating system-wide cross-talk between tissues as well as the far-reaching benefits of endurance exercise in the body. Such data provide a meaningful platform to build on to understand the intricate whole-body benefits of exercise.

## Figures and Tables

**Table 1:** Body, heart, and skeletal muscle weights in sedentary and exercise-trained rats. *N* = 12 per group. Values are expressed as mean values ± SE. * *P* < 0.05 vs. sedentary.

Weights	Group	
-	Sedentary	Exercise Trained
Body, (g)	392.5 ± 13.0	361.8 ± 8.1
Heart, (mg)	1205 ± 25	1208 ± 24
Soleus, (mg)	149.9 ± 7.2	151.9 ± 8.8
Plantaris, (mg)	426.6 ± 15.4	396.9 ± 16.7
Heart/Body, (mg/g)	3.10 ± 0.09	3.34 ± 0.05 *
Soleus/Body, (mg/g)	0.38 ± 0.01	0.42 ± 0.02
Plantaris/Body, (mg/g)	1.09 ± 0.05	1.10 ± 0.04

**Table 2:** Summary of *t*-test significantly altered metabolites across skeletal muscle, liver, serum, and heart in exercise trained animals compared to sedentary. Increased (↑) Decreased (↓). *Italics: metabolite found in more than one tissue*.

Significantly Altered Plantaris Metabolites with Exercise (*t*-Test, Figure 2C)	Significantly Altered Liver Metabolites with Exercise (*t*-Test, Figure 5C)	Significantly Altered Soleus Metabolites with Exercise (*t*-Test, Figure S3C,S5A)	Significantly Altered Serum Metabolites with Exercise (*t*-Test, Figure S4C,S5B)	Significantly Altered Heart Metabolites with Exercise (*t*-Test, Figure S7C)
C13 Hydrocarbon (↓)	-	-	-	-
Inosine/Adenosine (↑)	-	-	-	-
Fructose-6-Phosphate (↑)	-	-		-
Glucose-6-Phosphate or Similar (↑)	-	-	-	-
*Citric Acid/Isocitric Acid* (↑)	*Citric Acid/Isocitric Acid* (↓)	-	-	-
2-Aminoadipic Acid (↑)	-	-	-	-
Heptadecanoic Acid/Octadecanol (↑)	-	-	-	-
Stearic acid (↑)	-	-	-	-
*alpha-Tocopherol* (↑)	-	-	*alpha-Tocopherol* (↓)	-
*Linoleic Acid* (↑)	*Linoleic Acid* (↑)	-	-	-
Oleic Acid (↑)	-	-	-	-
-	Aldopentoses (↑)	-	-	-
-	-	Glycine (↓)	-	-
-	-	-	-	Malic acid (↑)
-	-	-	-	Serine (↑)
-	-	-	-	Aspartic acid (↓)
-	-	-	-	Myoinositol (↑)
-	-	-	-	Glutamine (↓)
-	-	-	-	Gluconic acid-6-phosphate (↑)
-	-	-	-	Glutamic acid (↑)
-	-	-	-	Pyrophosphate (↑)
-	-	-	-	Campesterol (↑)
-	-	-	-	Phosphoric acid (↑)
-	-	-	-	Creatinine (↑)

## Supplementary Materials

The following are available online at www.mdpi.com/2218-1989/7/3/40/s1, Figure S1: Non-targeted metabolomics analysis of exercise-trained and sedentary control rat plantaris muscle, Figure S2: Non-targeted metabolomics analysis of exercise-trained and sedentary control rat liver, Figure S3: Non-targeted metabolomics analysis of exercise-trained and sedentary control rat soleus muscle, Figure S4: Non-targeted metabolomics analysis of exercise-trained and sedentary control rat serum, Figure S5: Pathway analysis of *t*-test significant metabolites in exercise-trained soleus muscle and serum, Figure S6: Non-targeted metabolomics analysis of exercise-trained and sedentary control rat heart, Figure S7: Non-targeted metabolomics analysis of exercise-trained and sedentary control rat heart, Figure S8: Pathway analysis of *t*-test significant metabolites identified by GC-MS in exercise-trained heart, Figure S9: *t*-Test significant metabolites from heart, Table S1: Non-targeted metabolomics performed on exercise trained (or sedentary) rat.

## References

[1] Yang X., Li Y., Ren X., Xiong X., Wu L., Li J., Wang J., Gao Y., Shang H., Xing Y. (2017). Effects of exercise-based cardiac rehabilitation in patients after percutaneous coronary intervention: A meta-analysis of randomized controlled trials. Sci. Rep..

[2] Gordon B., Chen S., Durstine J.L. (2014). The effects of exercise training on the traditional lipid profile and beyond. Curr. Sports Med. Rep..

[3] Chorell E., Moritz T., Branth S., Antti H., Svensson M.B. (2009). Predictive metabolomics evaluation of nutrition-modulated metabolic stress responses in human blood serum during the early recovery phase of strenuous physical exercise. J. Proteom. Res..

[4] Gill J.M., Hardman A.E. (2003). Exercise and postprandial lipid metabolism: An update on potential mechanisms and interactions with high-carbohydrate diets. J. Nutr. Biochem..

[5] Gill J.M., Cooper A.R. (2008). Physical activity and prevention of type 2 diabetes mellitus. Sports Med..

[6] Gomez-Cabrera M.C., Domenech E., Vina J. (2008). Moderate exercise is an antioxidant: Upregulation of antioxidant genes by training. Free Radic. Biol. Med..

[7] Thompson P.D., Franklin B.A., Balady G.J., Blair S.N., Corrado D., Estes N.A., Fulton J.E., Gordon N.F., Haskell W.L., Link M.S. (2007). Exercise and acute cardiovascular events placing the risks into perspective: A scientific statement from the american heart association council on nutrition, physical activity, and metabolism and the council on clinical cardiology. Circulation.

[8] Lew J.K., Pearson J.T., Schwenke D.O., Katare R. (2017). Exercise mediated protection of diabetic heart through modulation of microrna mediated molecular pathways. Cardiovasc. Diabetol..

[9] Boccatonda A., Tripaldi R., Davi G., Santilli F. (2016). Oxidative stress modulation through habitual physical activity. Curr. Pharm. Des..

[10] Idorn M., Thor Straten P. (2017). Exercise and cancer: From “healthy” to “therapeutic”?. Cancer Immunol. Immunother..

[11] Idorn M., Hojman P. (2016). Exercise-dependent regulation of NK cells in cancer protection. Trends Mol. Med..

[12] Li T., Wei S., Shi Y., Pang S., Qin Q., Yin J., Deng Y., Chen Q., Wei S., Nie S. (2016). The dose-response effect of physical activity on cancer mortality: Findings from 71 prospective cohort studies. Br. J. Sports Med..

[13] Mielcarek M., Isalan M. (2015). A shared mechanism of muscle wasting in cancer and huntington’s disease. Clin. Transl. Med..

[14] Pescatello L.S., Franklin B.A., Fagard R., Farquhar W.B., Kelley G.A., Ray C.A., American College of Sports Medicine position stand (2004). Progression models in resistance training for healthy adults. Med. Sci. Sports Exerc..

[15] Bray M.S., Hagberg J.M., Perusse L., Rankinen T., Roth S.M., Wolfarth B., Bouchard C. (2009). The human gene map for performance and health-related fitness phenotypes: The 2006–2007 update. Med. Sci. Sports Exerc..

[16] Yan B., A J., Wang G., Lu H., Huang X., Liu Y., Zha W., Hao H., Zhang Y., Liu L. (2009). Metabolomic investigation into variation of endogenous metabolites in professional athletes subject to strength-endurance training. J. Appl. Physiol..

[17] Burniston J.G. (2008). Changes in the rat skeletal muscle proteome induced by moderate-intensity endurance exercise. Biochim. Biophys. Acta.

[18] Fazelzadeh P., Hangelbroek R.W., Tieland M., de Groot L.C., Verdijk L.B., van Loon L.J., Smilde A.K., Alves R.D., Vervoort J., Muller M. (2016). The muscle metabolome differs between healthy and frail older adults. J. Proteom. Res..

[19] Huang C.C., Lin W.T., Hsu F.L., Tsai P.W., Hou C.C. (2010). Metabolomics investigation of exercise-modulated changes in metabolism in rat liver after exhaustive and endurance exercises. Eur. J. Appl. Physiol..

[20] Feger B.J., Starnes J.W. (2013). Exercise alters the regulation of myocardial Na^+^/H^+^ exchanger-1 activity. Am. J. Physiol. Regul. Integr. Comp. Physiol..

[21] Nelson M.J., Harris M.B., Boluyt M.O., Hwang H.S., Starnes J.W. (2011). Effect of *N*-2-mercaptopropionyl glycine on exercise-induced cardiac adaptations. Am. J. Physiol. Regul. Integr. Comp. Physiol..

[22] Mitchell C.R., Harris M.B., Cordaro A.R., Starnes J.W. (2002). Effect of body temperature during exercise on skeletal muscle cytochrome *c* oxidase content. J. Appl. Physiol..

[23] Huffman K.M., Koves T.R., Hubal M.J., Abouassi H., Beri N., Bateman L.A., Stevens R.D., Ilkayeva O.R., Hoffman E.P., Muoio D.M. (2014). Metabolite signatures of exercise training in human skeletal muscle relate to mitochondrial remodelling and cardiometabolic fitness. Diabetologia.

[24] Bentley D.J., Ackerman J., Clifford T., Slattery K.S., Lamprecht M. (2014). Acute and chronic effects of antioxidant supplementation on exercise performance. Antioxidants in Sport Nutrition.

[25] Reid M.B., Stokic D.S., Koch S.M., Khawli F.A., Leis A.A. (1994). *N*-acetylcysteine inhibits muscle fatigue in humans. J. Clin. Investig..

[26] Watson T.A., MacDonald-Wicks L.K., Garg M.L. (2005). Oxidative stress and antioxidants in athletes undertaking regular exercise training. Int. J. Sport Nutr. Exerc. Metab..

[27] Reid M.B. (2001). Invited review: Redox modulation of skeletal muscle contraction: What we know and what we don’t. J. Appl. Physiol..

[28] Labazi M., McNeil A.K., Kurtz T., Lee T.C., Pegg R.B., Angeli J.P., Conrad M., McNeil P.L. (2015). The antioxidant requirement for plasma membrane repair in skeletal muscle. Free Radic. Biol. Med..

[29] Howard A.C., McNeil A.K., McNeil P.L. (2011). Promotion of plasma membrane repair by vitamin E. Nat. Commun..

[30] Dudley G.A., Abraham W.M., Terjung R.L. (1982). Influence of exercise intensity and duration on biochemical adaptations in skeletal muscle. J. Appl. Physiol. Respir. Environ. Exerc. Physiol..

[31] Bowles D.K., Farrar R.P., Starnes J.W. (1992). Exercise training improves cardiac function after ischemia in the isolated, working rat heart. Am. J. Physiol..

[32] Ariano M.A., Armstrong R.B., Edgerton V.R. (1973). Hindlimb muscle fiber populations of five mammals. J. Histochem. Cytochem..

[33] Banerjee R., Bultman S.J., Holley D., Hillhouse C., Bain J.R., Newgard C.B., Muehlbauer M.J., Willis M.S. (2015). Non-targeted metabolomics of Brg1/Brm double-mutant cardiomyocytes reveals a novel role for swi/snf complexes in metabolic homeostasis. Metabolomics.

[34] Guler A.T., Waaijer C.J., Palmblad M. (2016). Scientific workflows for bibliometrics. Scientometrics.

[35] Rocca-Serra P., Salek R.M., Arita M., Correa E., Dayalan S., Gonzalez-Beltran A., Ebbels T., Goodacre R., Hastings J., Haug K. (2016). Data standards can boost metabolomics research, and if there is a will, there is a way. Metabolomics.

[36] Sud M., Fahy E., Cotter D., Azam K., Vadivelu I., Burant C., Edison A., Fiehn O., Higashi R., Nair K.S. (2016). Metabolomics workbench: An international repository for metabolomics data and metadata, metabolite standards, protocols, tutorials and training, and analysis tools. Nucleic Acids Res..

[37] Xia J., Psychogios N., Young N., Wishart D.S. (2009). Metaboanalyst: A web server for metabolomic data analysis and interpretation. Nucleic Acids Res..

[38] Xia J., Sinelnikov I.V., Han B., Wishart D.S. (2015). MetaboAnalyst 3.0—Making metabolomics more meaningful. Nucleic Acids Res..

