# Supplementary material for: Exercise-Induced Alterations in Skeletal Muscle, Heart, Liver, and Serum Metabolome Identified by Non-Targeted Metabolomics Analysis
Source: Metabolites. 2017 Aug 8;7(3):40. doi: 10.3390/metabo7030040 (PMC5618325; doi:10.3390/metabo7030040)
Supplement: Supplementary file 1 [file metabolites-07-00040-s001.zip › metabolites-214484-publish-supplemtent-figures.pdf]

Supplementary Materials: Exercise-Induced Alterations in Skeletal Muscle, Heart, Liver, and Serum Metabolome Identified by Non-Targeted Metabolomics Analysis

Joseph W. Starnes [1,†], Traci L. Parry [2,3,†], Sara K. O'Neal [4], James R. Bain [4,5], Michael J. Muehlbauer [5], Aubree Honcoop [6], Amro Ilaiwy [4,5], Peter M. Christopher [1], Cam Patterson [7], Monte S. Willis [2,3,8]

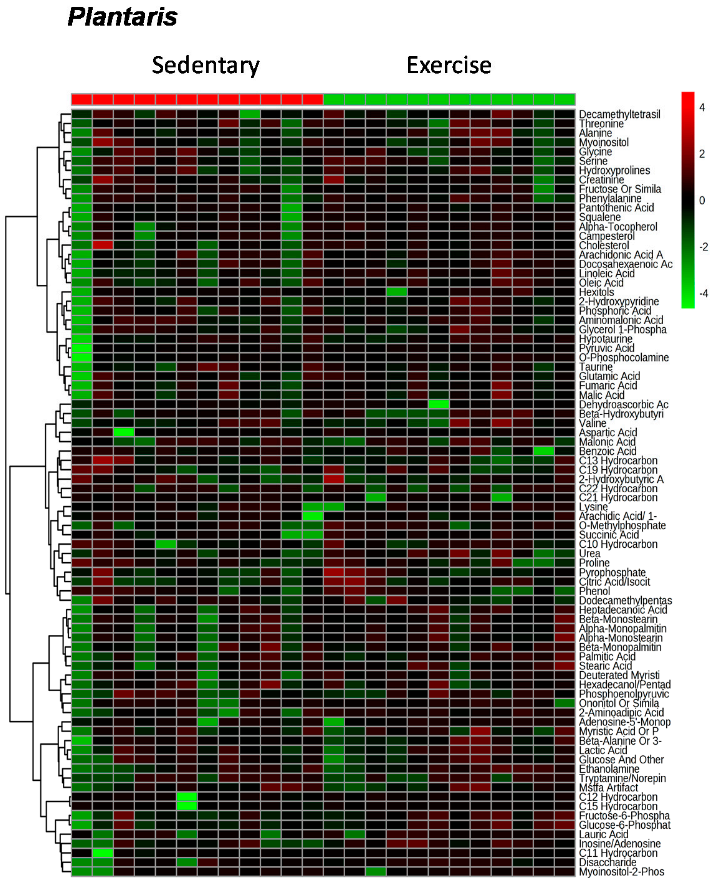

Figure S1. Non-targeted metabolomics analysis of exercise-trained and sedentary control rat plantaris muscle. Heatmap of metabolites identified by GC-MS in the heart. $N = 12$/group.

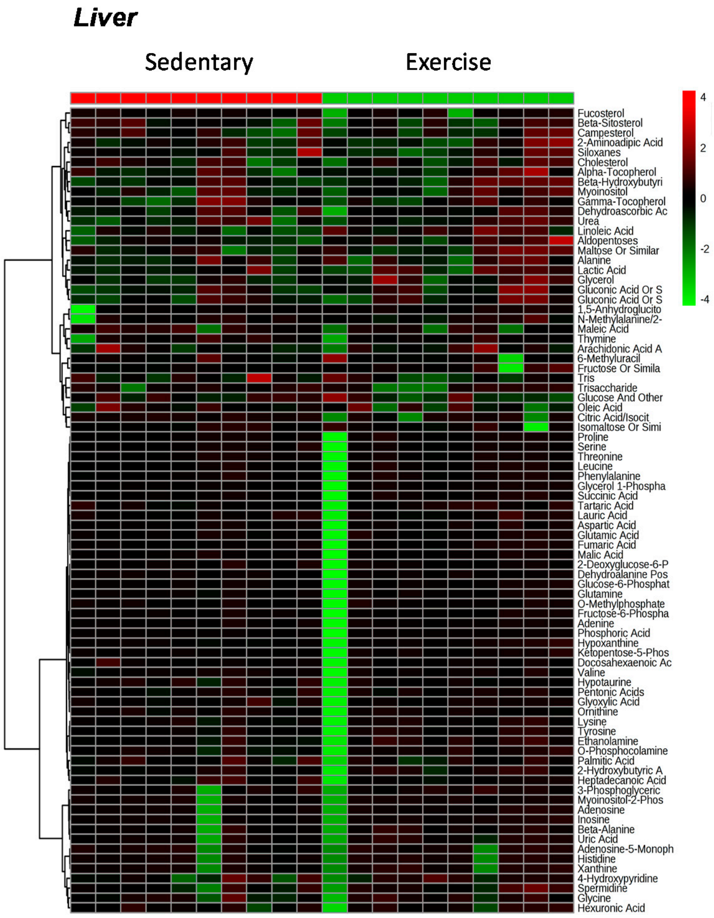

Figure S2. Non-targeted metabolomics analysis of exercise-trained and sedentary control rat liver. Heatmap of metabolites identified by GC-MS in the heart. $N = 12$/group.

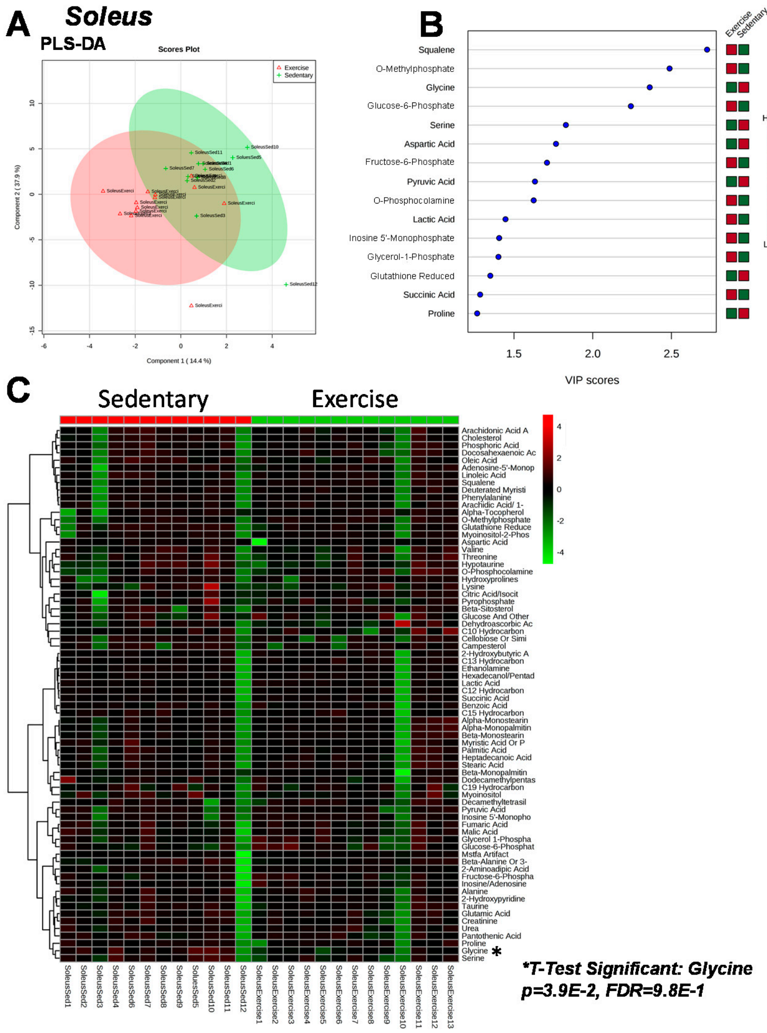

Figure S3. Non-targeted metabolomics analysis of exercise-trained and sedentary control rat soleus muscle. (**A**) Principal components analysis using PLS-DA. (**B**) Variable importance in projection (VIP) scores. (**C**) Heatmap of metabolites identified by GC-MS in the heart. **P* < 0.05. *N* = 12/group.

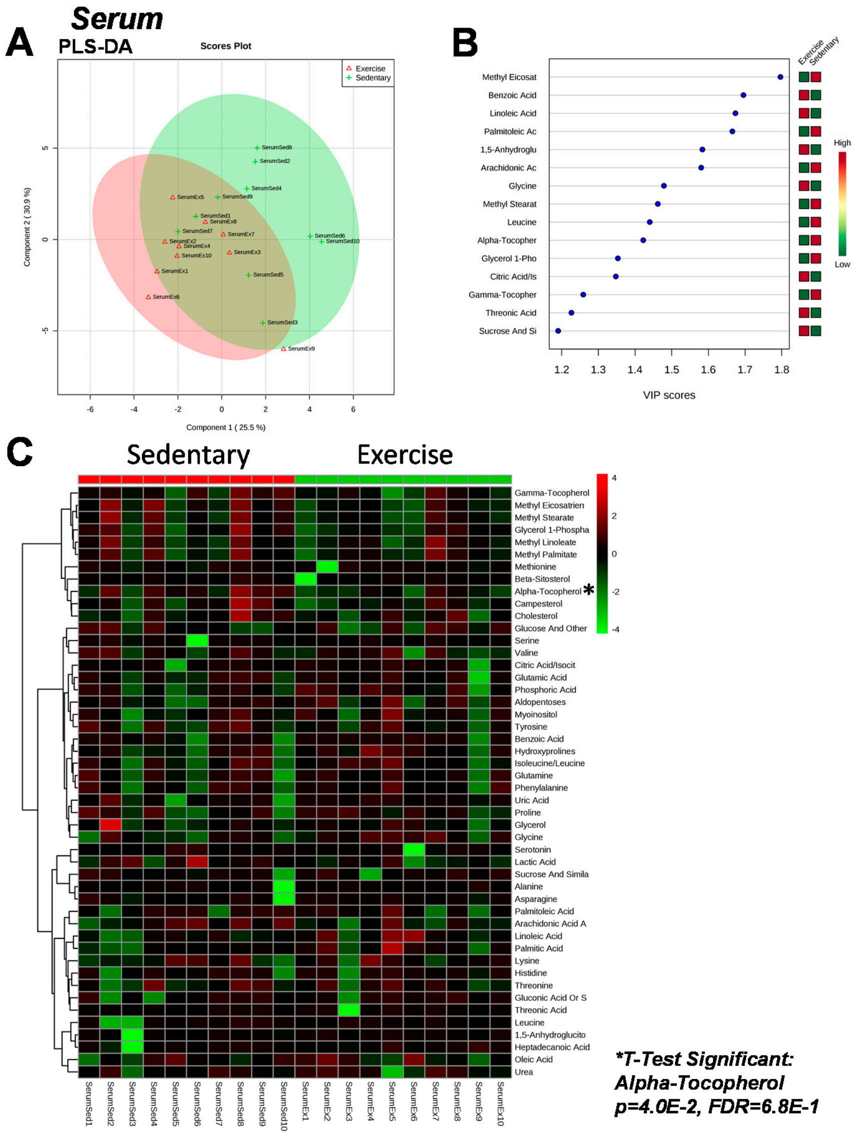

Figure S4. Non-targeted metabolomics analysis of exercise-trained and sedentary control rat serum. (**A**) Principal components analysis using PLS-DA. (**B**) Variable importance in projection (VIP) scores. (**C**) Heatmap of metabolites identified by GC-MS in the heart. $^{*}P < 0.05$. $N = 12$/group.

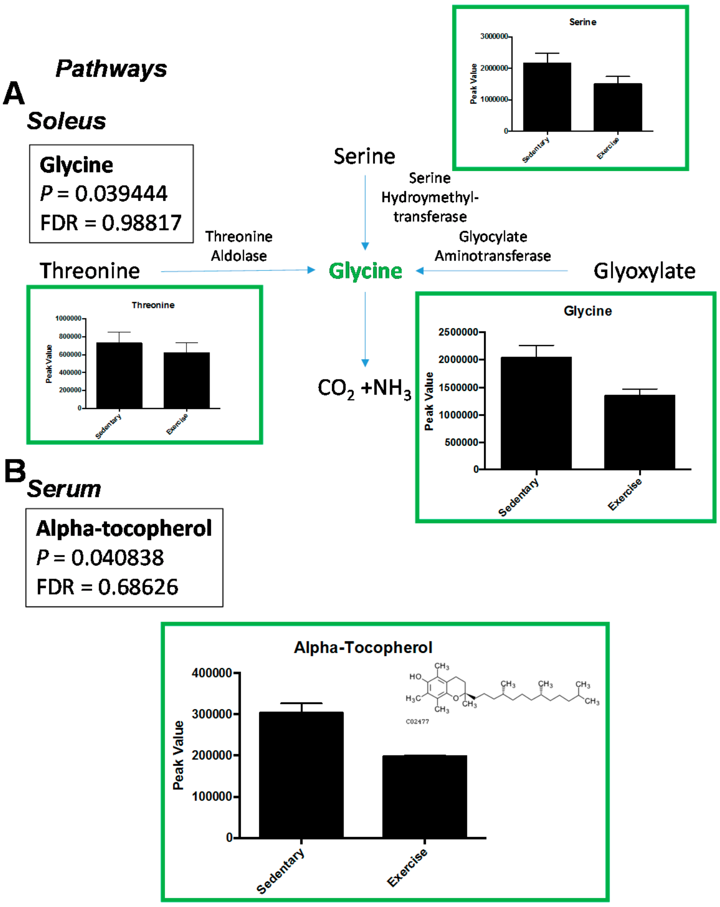

Figure S5. Pathway analysis of *t*-test significant metabolites in exercise-trained soleus muscle and serum. (**A**) Alterations in soleus muscle glycine (and other non-significant identified metabolites) identified in the pathway. (**B**) Alterations in serum alpha-tocopherol. Data represent mean ± SEM. *$P < 0.05$. $N = 12$/group.

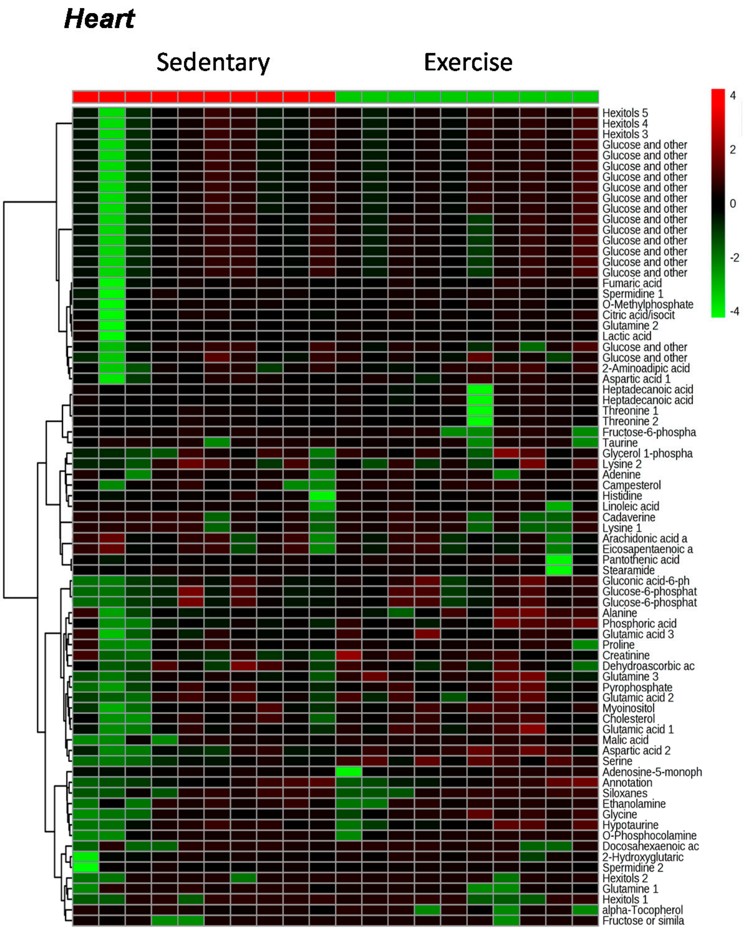

Figure S6. Non-targeted metabolomics analysis of exercise-trained and sedentary control rat heart. Heatmap of metabolites identified by GC-MS in the heart. N = 12/group.

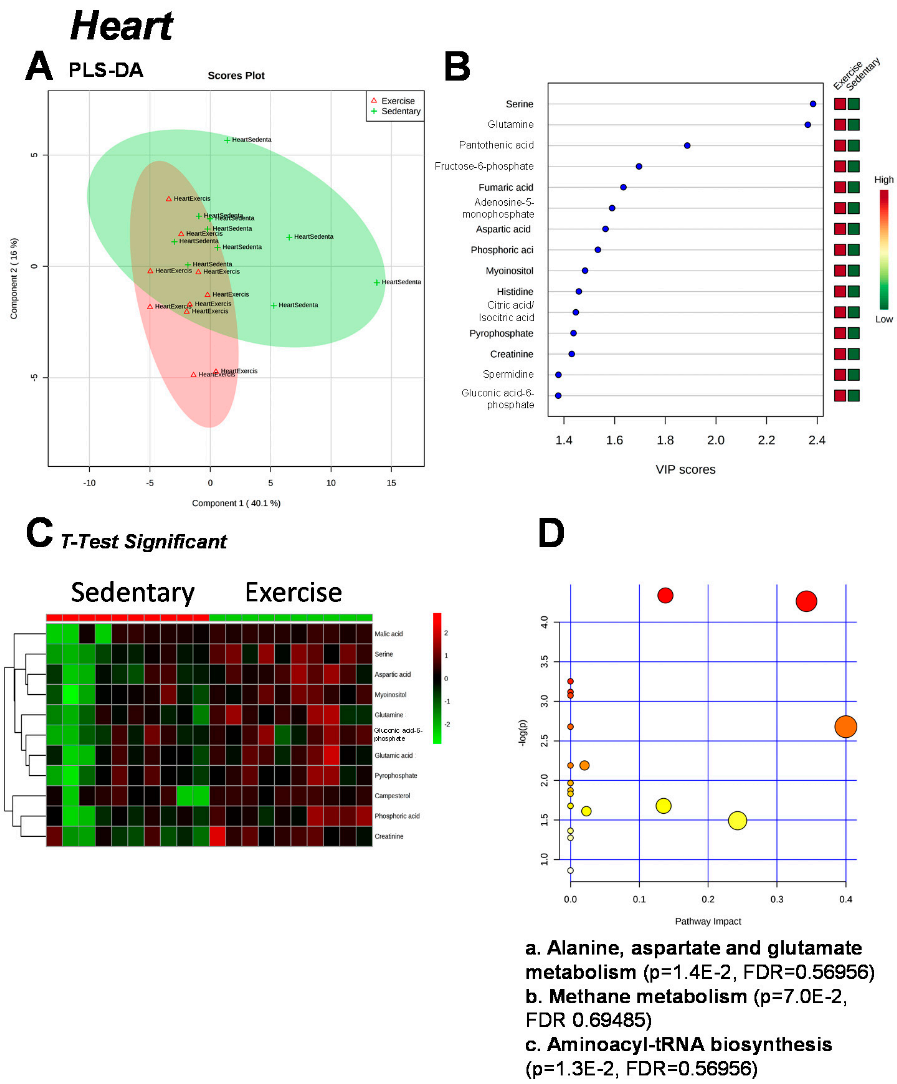

Figure S7. Non-targeted metabolomics analysis of exercise-trained and sedentary control rat heart. (**A**) Principal components analysis using PLS-DA. (**B**) Variable importance in projection (VIP) scores. (**C**) *t*-Test significant heatmap of metabolites identified by GC-MS in the heart. (**D**) Pathway analysis using *t*-test significant metabolites identifying alanine, aspartate, and glutamate metabolism as highly enriched. $*P < 0.05$. $N = 12$/group.

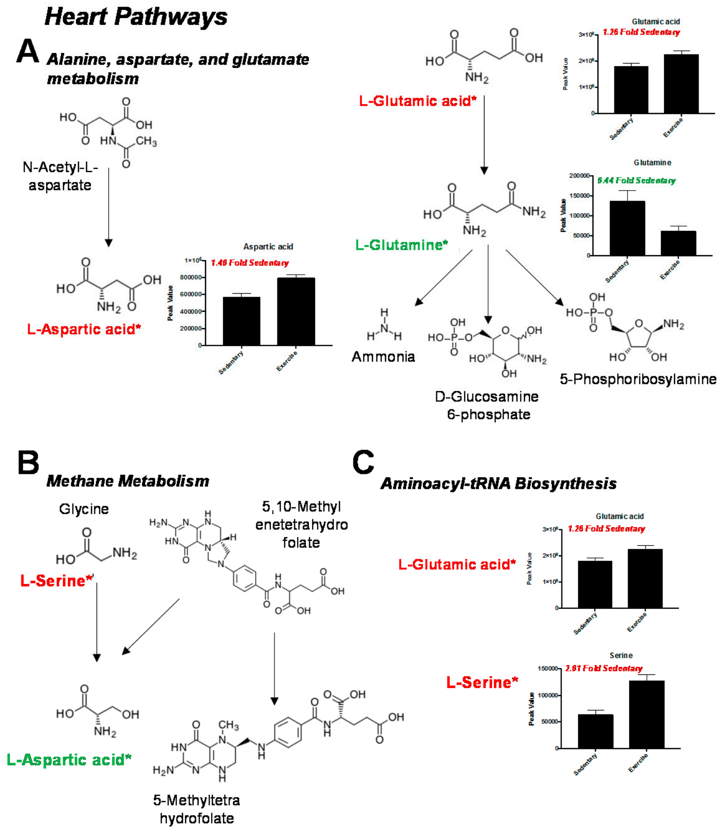

Figure S8. Pathway analysis of t-test significant metabolites identified by GC-MS in exercise-trained heart. (**A**) Alanine, aspartate, and glutamate metabolism. (**B**) Methane metabolism. (**C**) Aminoacyl-tRNA Biosynthesis. Data represent mean ± SEM. *$P < 0.05$. $N = 12$/group.

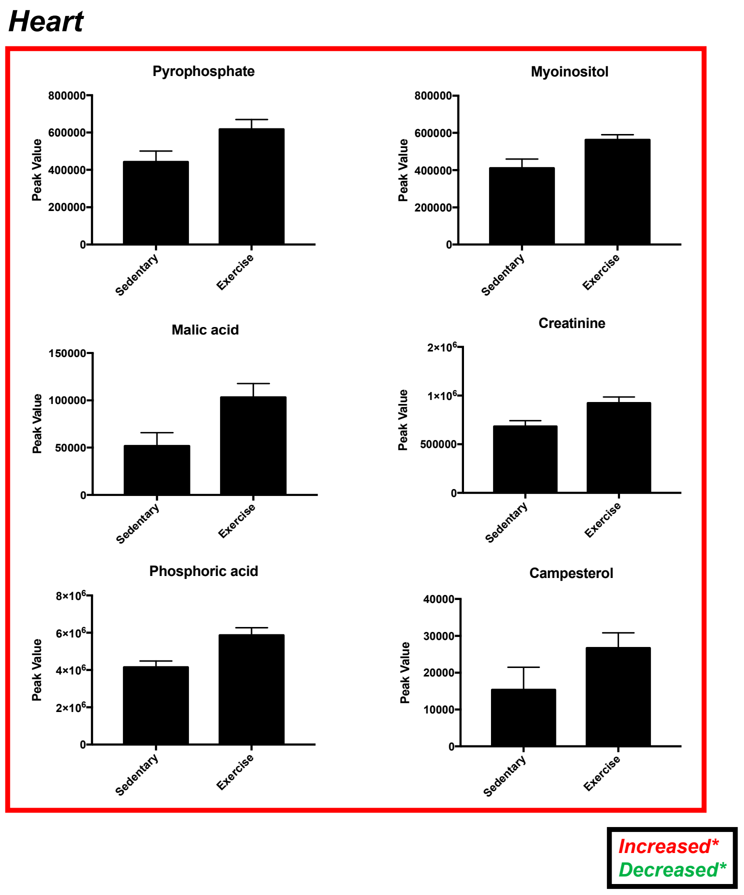

Figure S9. *t*-Test significant metabolites from heart. Data represent mean ± SEM. *$P < 0.05$. $N = 12$/group.
